# Supplementary material for: Engineering brain activity patterns by neuromodulator polytherapy for treatment of disorders
Source: Nat Commun. 2019 Jun 13;10:2620. doi: 10.1038/s41467-019-10541-1 (PMC6565674; doi:10.1038/s41467-019-10541-1)
Supplement: Supplementary file 1 — Supplementary Information [file 41467_2019_10541_MOESM1_ESM.pdf]

Engineering brain activity patterns by neuromodulator polytherapy for treatment of disorders

Ghannad-Rezaie, Eimon, Wu, Yanik

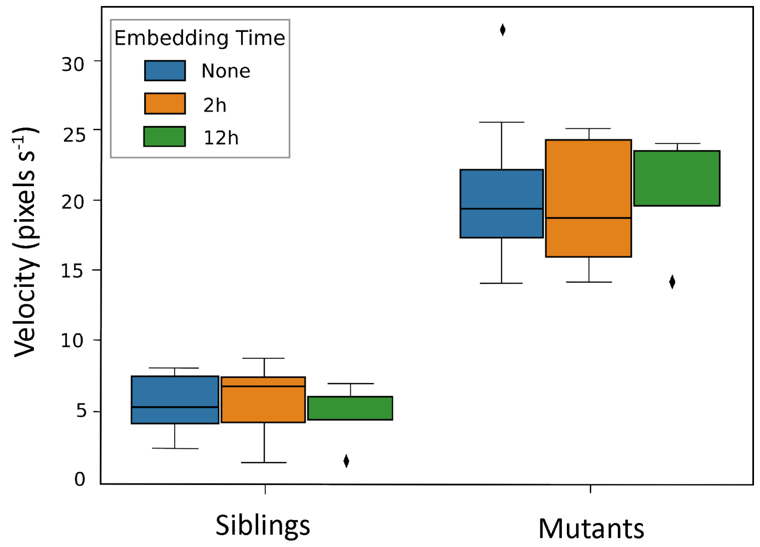

Supplementary Figure 1: Agar-embedded zebrafish larvae remain viable and healthy for up to 12 hours. As described in **Methods**, larvae are embedded in the center of a dual layer agarose cylinders consisting of a 1.3% ultra-low gelling temperature agarose core (which solidifies at 25°C) surrounded by shell of 2% low gelling temperature agarose (which solidifies at 55°C). Embedded larvae (n=10 per experimental condition) are then incubated for either 2 hours (orange) or 12 hours (green) in standard embryo medium, manually released from the agar, and behaviorally assessed using an automated video tracking platform. Agar-embedded wild-type larvae and scn1lab mutants both exhibit normal levels of locomotor activity when compared to non-embedded controls from the same clutch (blue). Larvae are recorded in a 96-well plate and exposed to light stimuli as outlined in **Fig. 2a**. Mean swimming velocities (pixels per second) are calculated during 5 second intervals following each light pulse. Tops and bottoms of each box represent the 1st and 3rd quartiles. Whiskers are drawn from the ends of the interquartile ranges (IQR) to the outermost data point that falls within ±1.5 times the IQR. The line in the middle of each box is the sample median. Source data are provided as a Source Data file.

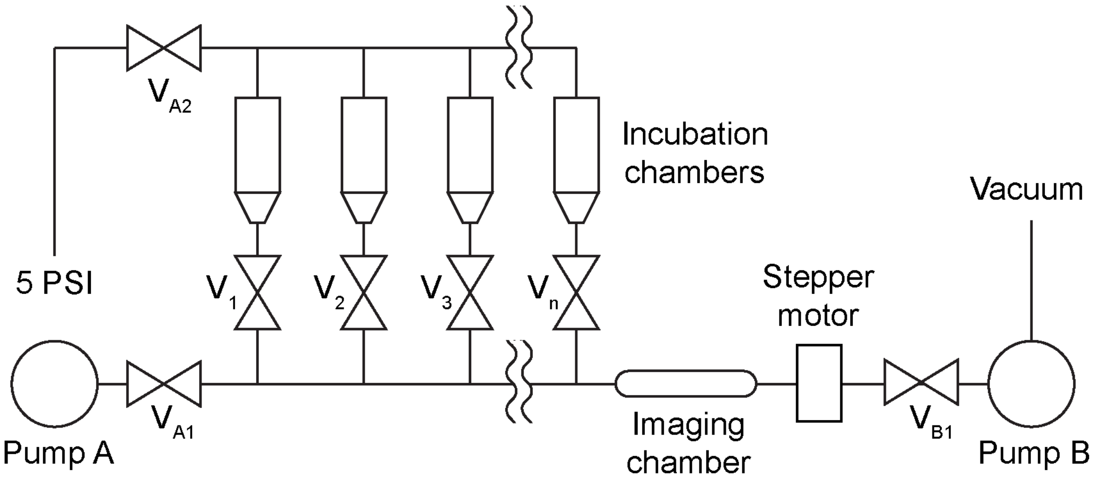

Supplementary Figure 2: Schematic diagram of fluidic components. The fluidic system is used for fast and precise placement of larva inside imaging chamber from multiple drug incubation chambers. The system consists of two computerized syringe pumps and a series of pinch valves to control the flow between the pumps. To load larvae from one of the incubation reservoirs into the imaging chamber, the glass capillary is first flushed to remove any residual liquid from the previous imaging step. This is done by opening pinch valves A1 and B1 and transferring 1 mL of E3 medium from syringe pump A into syringe pump B. Both pinch valves are then closed and the E3 medium is expelled from syringe pump B into a vacuum flask. To load larvae from the n-th incubation reservoir in the array, we open pinch valves V_n, A2, and B1. Syringe pump B then drains liquid from the n-th reservoir until the first larva is in the imaging chamber. When the larva is in the place, pinch valves V_n, A2, and B1 are closed. After the imaging session is completed, all 3 valves are opened and continue draining the reservoir until the next larva is in the imaging chamber. This process is repeated until all larvae contained in the n-th incubation reservoir have been imaged, at which point pinch valves are opened and the larvae are transferred back into the incubation reservoir using syringe pump B.

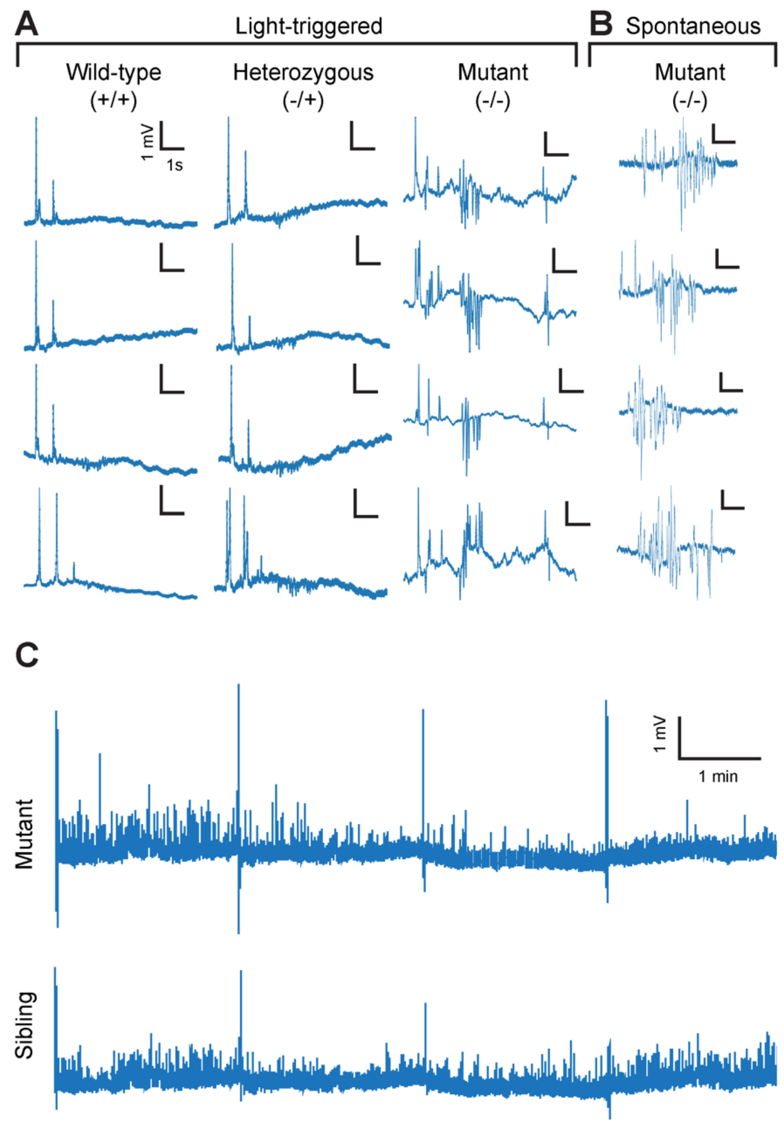

Supplementary Figure 3: Light stimuli trigger seizures in zebrafish larvae with scn1lab mutations. (**a**) LFP recordings from the forebrains of scn1lab^{s552} homozygous mutant (-/-) larvae and age-matched heterozygous mutant (-/+) and wild-type (+/+) sibling controls in response to a single light stimulus consisting of two consecutive 500 millisecond light pulses separated by 1 second of dark. (**b**) LFP recordings illustrating spontaneous seizures detected by our automated seizure detection algorithm in scn1lab^{s552} homozygous mutant (-/-) larvae. (**c**) Representative LFP recordings from a mutant and a sibling control showing response to multiple light stimuli presented every 2 minutes over the course of an 8-minute recording session. (**a-b**) Scale bars show 1 second (x-axis) and 1 mV (y-axis). (**c**) Scale bars show 1 minute (x-axis) and 1 mV (y-axis).

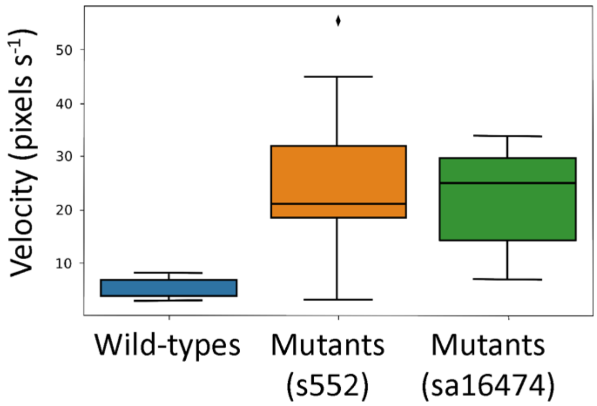

Supplementary Figure 4: Light stimuli trigger seizure-like locomotor activity in scn1lab mutant larvae. Box-and-whisker plots show mean swimming velocity (pixels per second) for wild-type larvae (blue; n=10) and homozygous mutants from the s552 line (orange; n=10) and the sa16474 line (green; n=10). Larvae are recorded in a 96-well plate using an automated tracking platform. Each light stimulus consists of two consecutive 500 millisecond light pulses separated by 1 second of darkness and each larva was subjected to 4 independent light stimuli. Mean swimming velocities (pixels per second) are calculated during 5 second intervals following each light pulse. Tops and bottoms of each box represent the 1st and 3rd quartiles. Whiskers are drawn from the ends of the interquartile ranges (IQR) to the outermost data point that falls within ±1.5 (SD) times the IQR. The line in the middle of each box is the sample median. Source data are provided as a Source Data file.

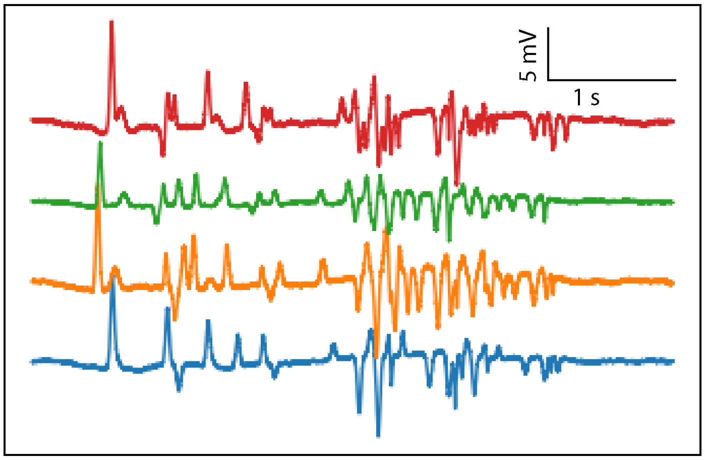

Supplementary Figure 5: Light-triggered seizures in paralyzed scn1lab mutant larvae. LFP recordings from the forebrains of scn1lab^{s552} homozygous mutant larvae at 5 dpf in response to light stimuli. In order to eliminate any light-triggered movement artifacts, larvae were embedded in ultra-low gelling temperature agarose containing 0.3 mg/ml of pancuronium bromide, a paralyzing agent. Light stimuli consisted of two consecutive 500 millisecond light pulses separated by 1 second of dark. Scale bars show 1 second (x-axis) and 5 mV (y-axis).

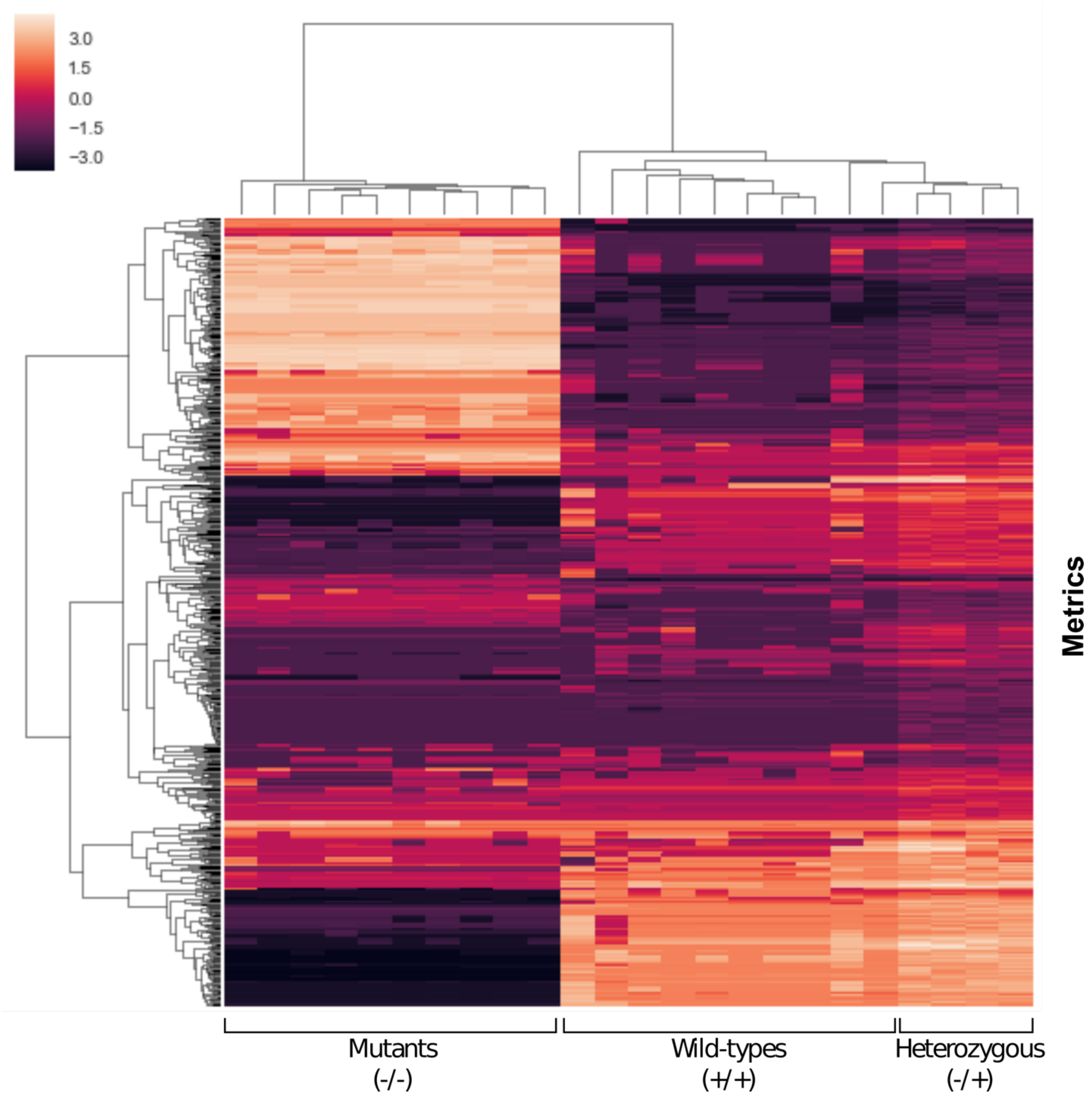

Supplementary Figure 6: Zebrafish with scn1lab mutations show altered functional brain connectivity. GCaMP activity is recorded for 10 minutes in the absence of light stimuli using light-sheet microscopy. Seizure-inducing light stimuli are then applied every two minutes in an otherwise dark environment. Each stimulus consists of two consecutive 500 millisecond light pulses separated by 1 second of dark. GCaMP recordings are subdivided as shown in **Fig. 2a** into pre-stimulus, early post-stimulus, late post-stimulus states for subsequent analysis. For each individual larva, a 165-metric functional connectivity fingerprint is generated consisting of correlation coefficients between all 55 pairs of brain areas during each of the three activity states. Each square represents the correlation between active supervoxels detected in the brain areas being compared. Functional connectivity fingerprints are shown for homozygous scn1lab^{s552} mutants (-/-; n=10) and sibling controls that are either homozygous wild-type (+/+; n=10) or heterozygous for the scn1lab mutation (-/+; n=4). Fingerprints are analyzed by hierarchical

clustering, confirming that functional connectivity fingerprints from mutants differ substantially from wild-type and heterozygous sibling controls.

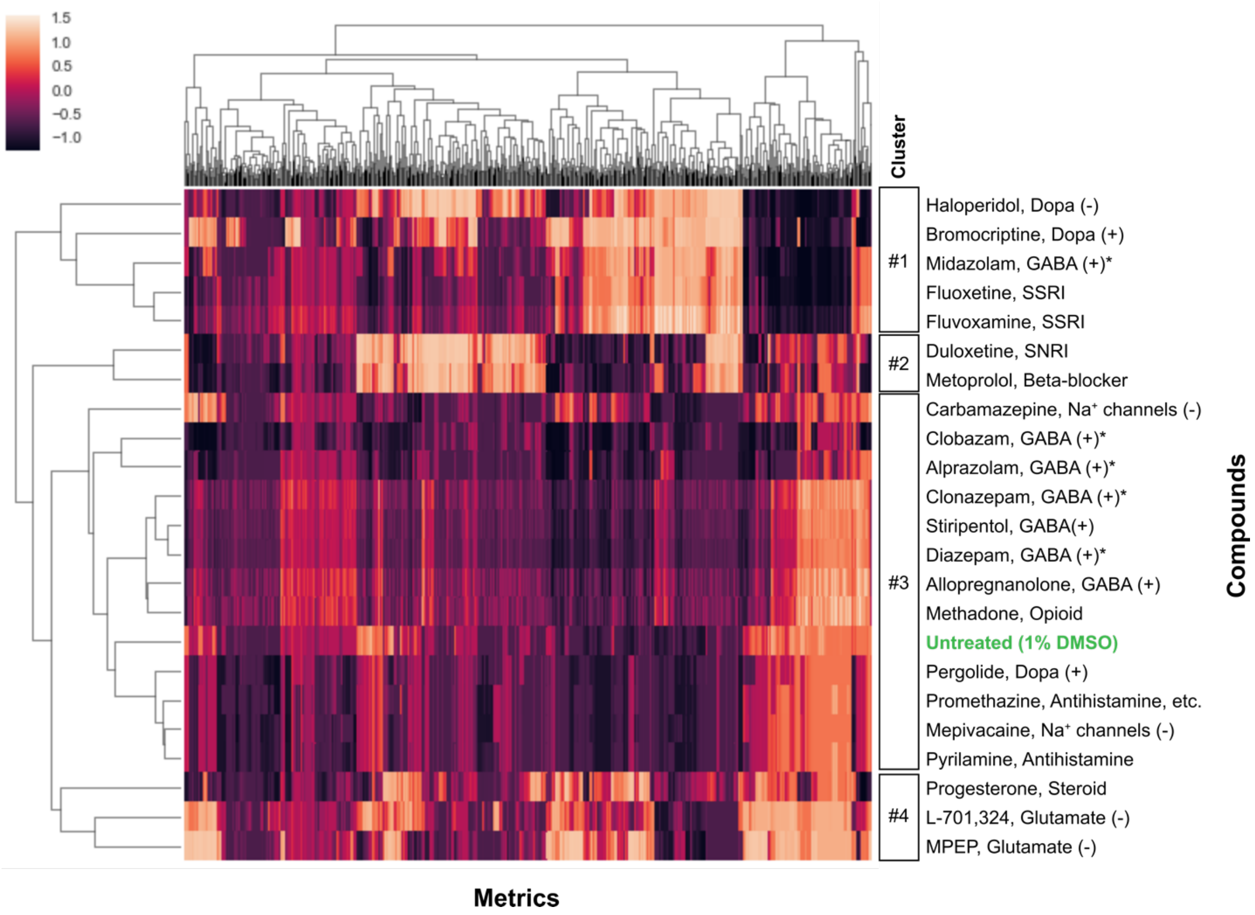

Supplementary Figure 7: Neuroactive drugs with differing mechanisms of action alter functional connectivity in distinct ways. GCaMP activity is recorded for 10 minutes in the absence of light stimuli using light-sheet microscopy. Seizure-inducing light stimuli are then applied every two minutes in an otherwise dark environment. Each stimulus consists of two consecutive 500 millisecond light pulses separated by 1 second of dark. GCaMP recordings are subdivided as shown in **Fig. 2a** into pre-stimulus, early post-stimulus, late post-stimulus states for subsequent analysis. For each compound in our screen, a 165-metric functional connectivity fingerprint is generated consisting of correlation coefficients between all 55 pairs of brain areas during each of the three activity states analyzed. Each square represents the average normalized correlation between active supervoxels detected in the brain areas being compared. A minimum of 5 larvae are analyzed per compound. This sample size is chosen to ensure sufficient statistical power to separate mutant larvae from sibling controls (unpaired Student's t-test, $p < 0.01$; normality of datasets was tested using the Jarque–Bera normality test). Functional connectivity fingerprinting is carried out at 4 hours post-exposure to the indicated compounds in 1% DMSO and fingerprints are analyzed by hierarchical clustering.

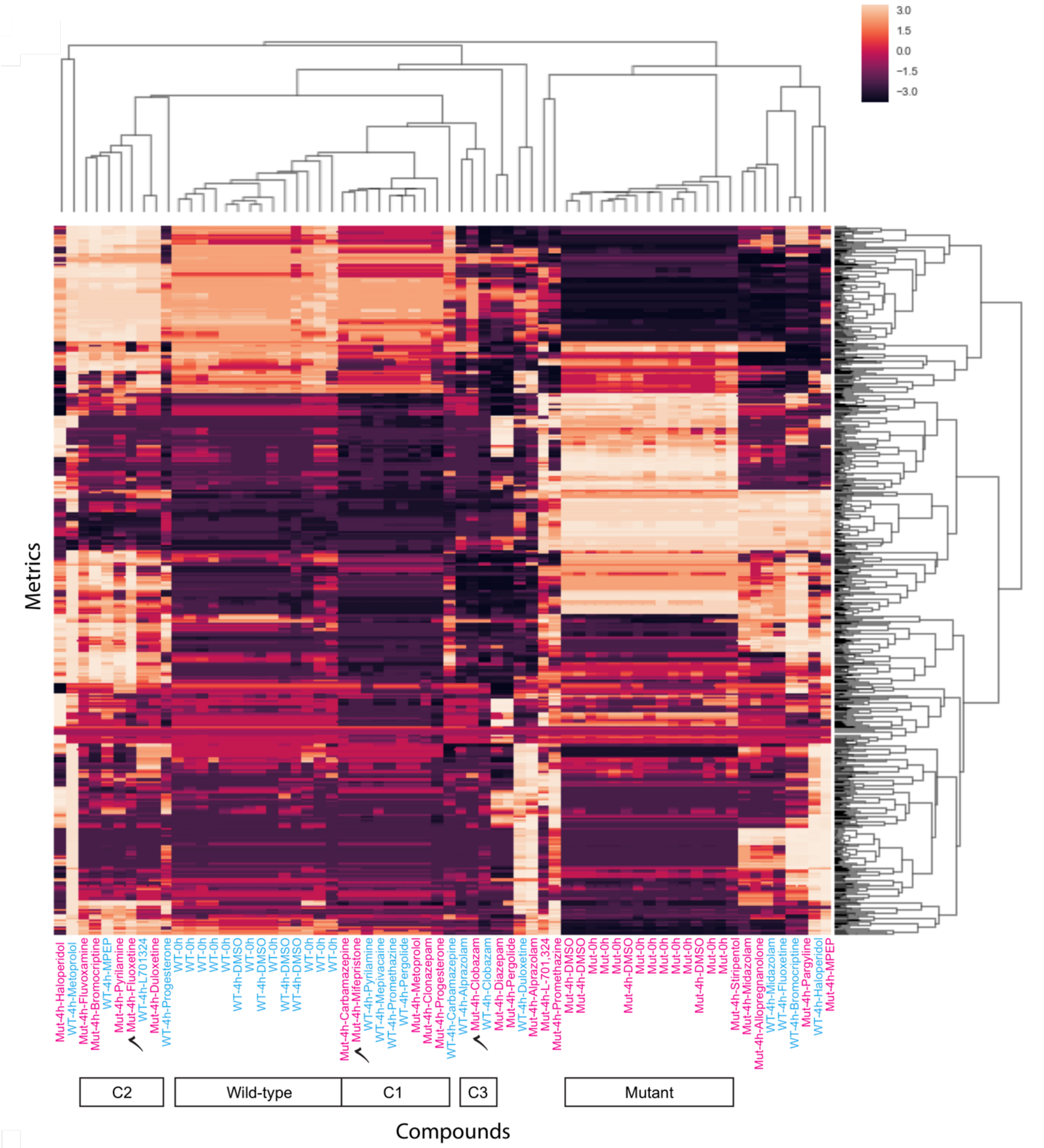

Supplementary Figure 8. High resolution brain connectivity fingerprint. The high resolution version of figure 2C.

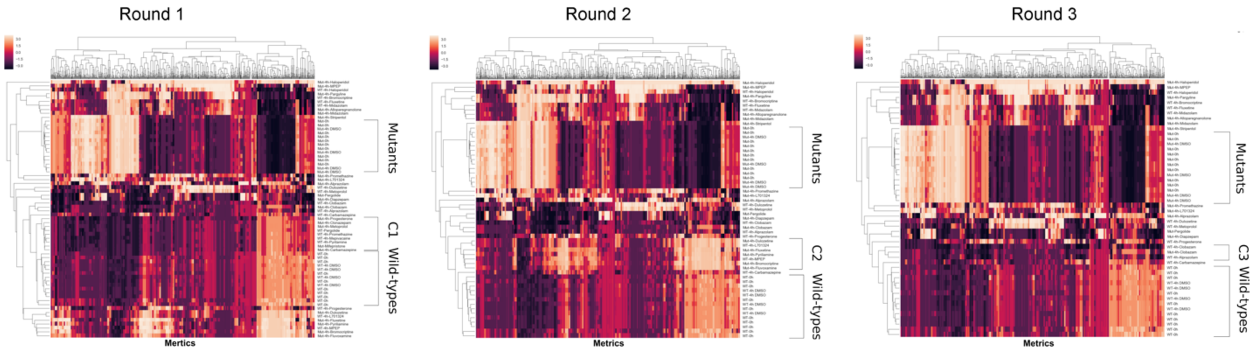

Supplementary Figure 9. Cluster identification and elimination. For each compound in our screen, a functional connectivity fingerprint is generated as described in **Fig. 2c**. Functional connectivity fingerprinting is carried out in both mutants (Mut) and sibling controls (WT) prior to compound addition and again at 4 hours post-exposure (4h). Prior to compound application, all untreated mutants and sibling controls localize to distinct clusters (indicated by mutant and wild-type brackets, respectively). In order to identify polytherapy candidates that are likely to normalize complementary facets of pathological connectivity, we utilize a selection strategy the incorporates multiple rounds of hierarchical cluster identification and elimination. In round 1, we identify the closest cluster to the wild-type cluster (C1). We then select the compound on C1 that is most effective at normalizing connectivity in scn1lab mutants (mifepristone). In round 2, we eliminate all compounds that localize to C1, perform a new round of clustering on the remaining compounds, and repeat the selection process to identify a second hit (fluoxetine). The entire process is repeated again in round 3 to identify a third hit (clobazam).

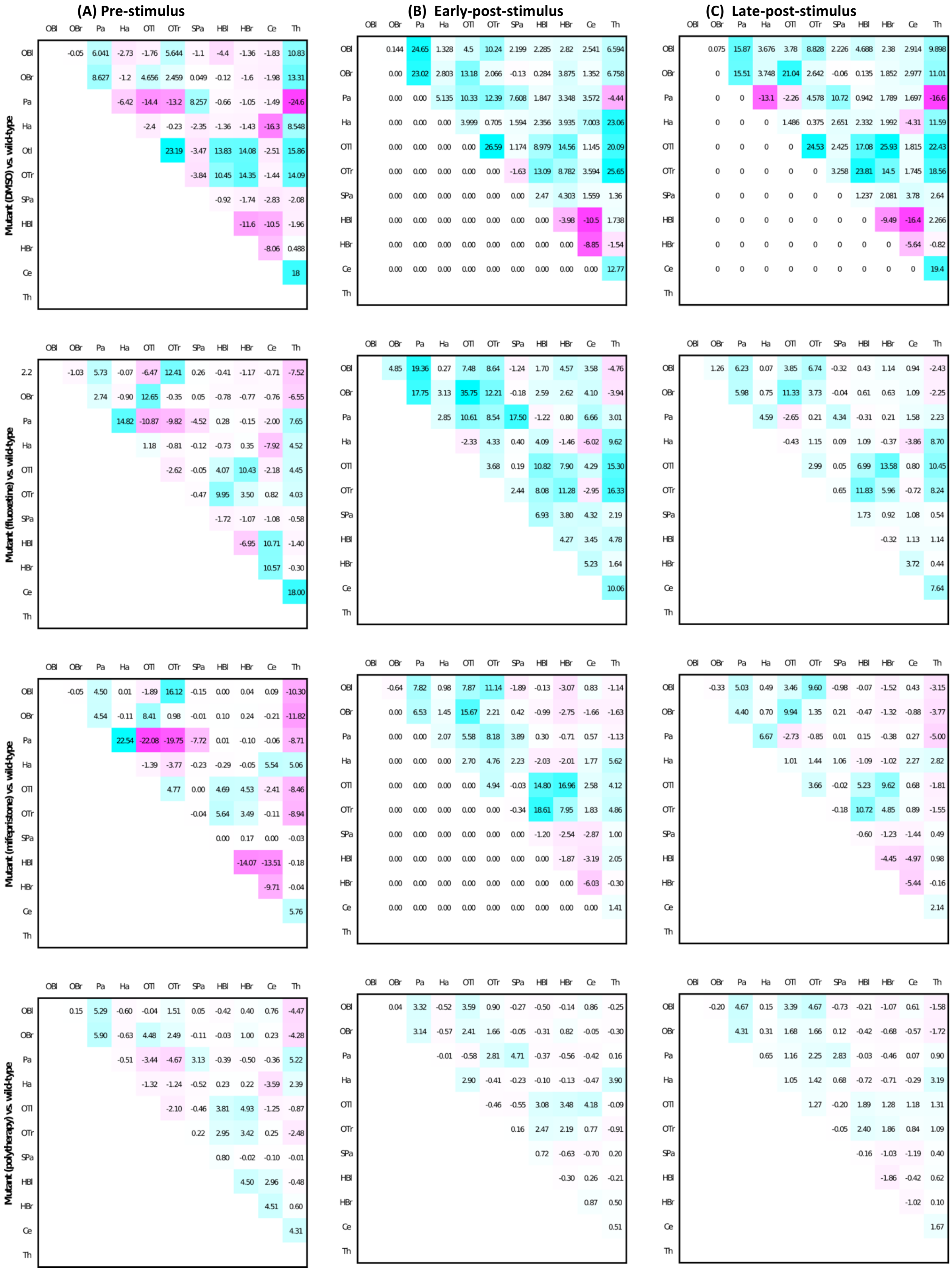

(A) Pre-stimulus
(B) Early-post-stimulus
(C) Late-post-stimulus
OBl OBr Pa Ha OTl OTr SPa HBl HBr Ce Th
Mutant (DMSO) vs. wild-type
Mutant (fluoxetine) vs. wild-type
Mutant (mifepristone) vs. wild-type
Mutant (polytherapy) vs. wild-type

Supplementary Figure 10: Polytherapy is more effective than monotherapy at normalizing functional brain connectivity in scn1lab mutants. Matrices show the percent increase (cyan) or decrease (magenta) in functional connectivity relative to wild-type sibling controls in scn1lab mutant larvae under the following conditions: (**row 1**) untreated mutants (1% DMSO), (**row 2**) mutants treated with fluoxetine alone at the 100% dose, (**row 3**) mutants treated with mifepristone alone at the 100% dose, and (**row 4**) mutants treated simultaneously with fluoxetine and mifepristone using one of the optimal polytherapy dose regimens (both drugs at 30%). Functional connectivity for each pair of brain areas is determined by calculating the average correlation between all active supervoxels within each area during **(a)** the pre-stimulus, **(b)** early-post-stimulus, and **(c)** late-post-stimulus states. For each compound, the highest dose (100%) corresponds to the concentration used in the initial light-sheet-based screen (see **Supplementary Table 1**). Ce, cerebellum; HBl, hindbrain left; HBr, hindbrain right; OBl, olfactory lobe left; OBr, olfactory lobe right; OTl, optical tectum, left; OTr, optical tectum, right; Pa, pallium; SPa, sub-pallium; Ha, Ha, habenula; Th, thalamus.

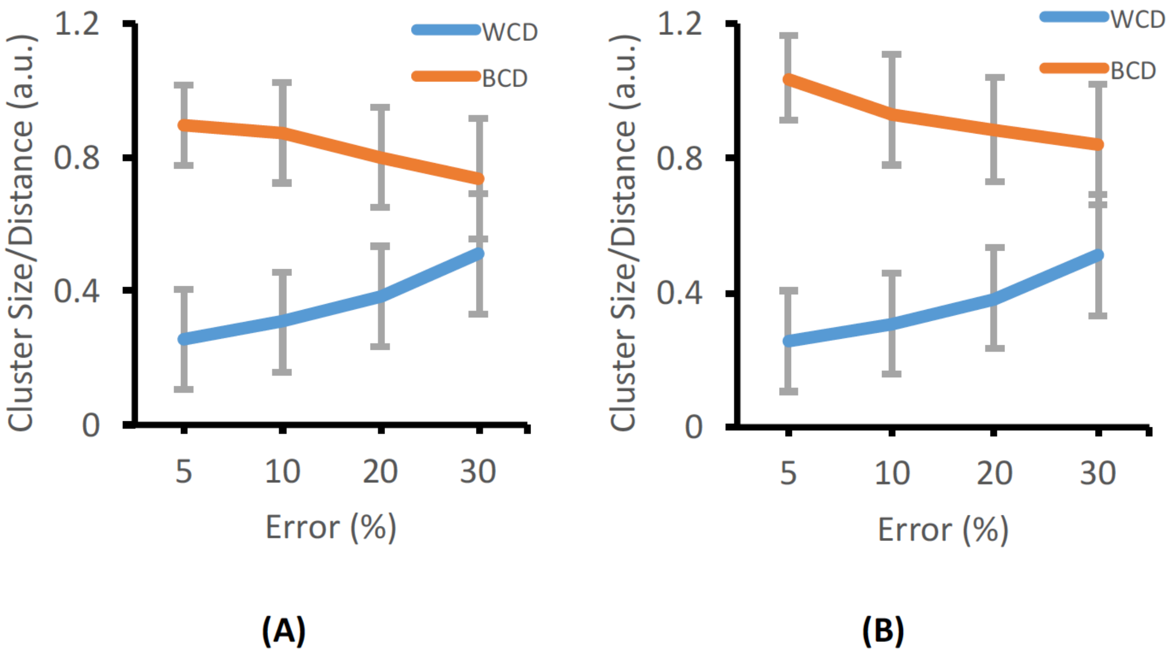

Supplementary Figure 11. Segmentation method is sufficiently accurate for the clustering algorithm. To demonstrate the segmentation is sufficiently accurate, we calculated the clusters in figure 2C after randomly modifying the segmentation such that the new segmentation has only 95% overlap with the original segmentation and therefore adding 5% simulated error to the segmentation results. We repeated this method with 10%, 20% and 30% segmentation error and measured the change in the clustering results. We measured the distance between **(a)** the original C1 and C2 clusters, **(b)** the original C1 and C3 clusters (BCD) and within the C1 cluster distance (WCD). Our results show more than 20% segmentation error in average affects the outcome of our clustering algorithm, while less than 10% average segmentation error does not affect our clustering at all. The graphs represent mean ±SD (n= 20). Source data are provided as a Source Data file.

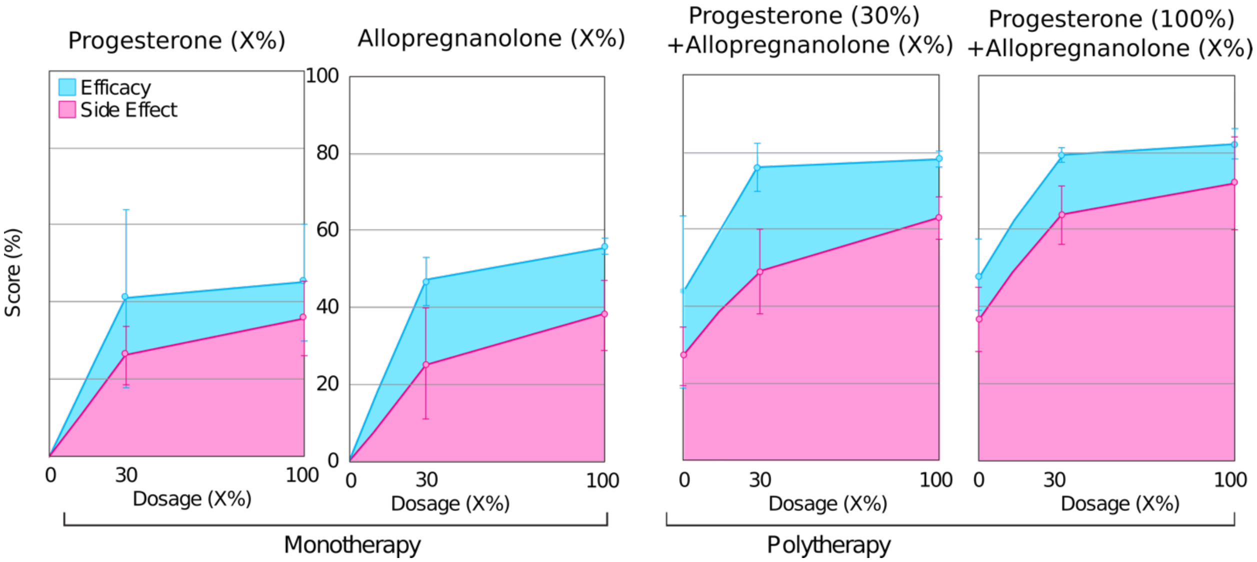

Supplementary Figure 12. Polytherapy based only on behavioral outcome shows higher side effects and lower efficacy. Dose-response data (at 0%, 30% and 100% of dosage) showing efficacy scores (cyan) and side effect scores (magenta) for two top behavioral hits with different MOAs from our previous work[33]: Allopregnanolone(GABA agonist) and progesterone(progesterone receptor agonist). Monotherapy and polythreapy over all investigated doses are shown at ~4 hours post-exposure. Allopregnanolone (100%) + progesterone (100%) combination gives 83% efficacy, which is not as good as mifepristone (30%) + fluoxetine (30%) (91% efficacy; **Fig. 3**), and also has a behavioral side effect score of 0.75 (compared to only 0.38 for the F+M combination; **Fig. 3**). Combining simply the top behavioral hits, even after selecting those with different known MOAs is not as good as the combination based on our connectivity analysis (in **Fig. 3**). The graphs represent mean ± SD (n = 5).

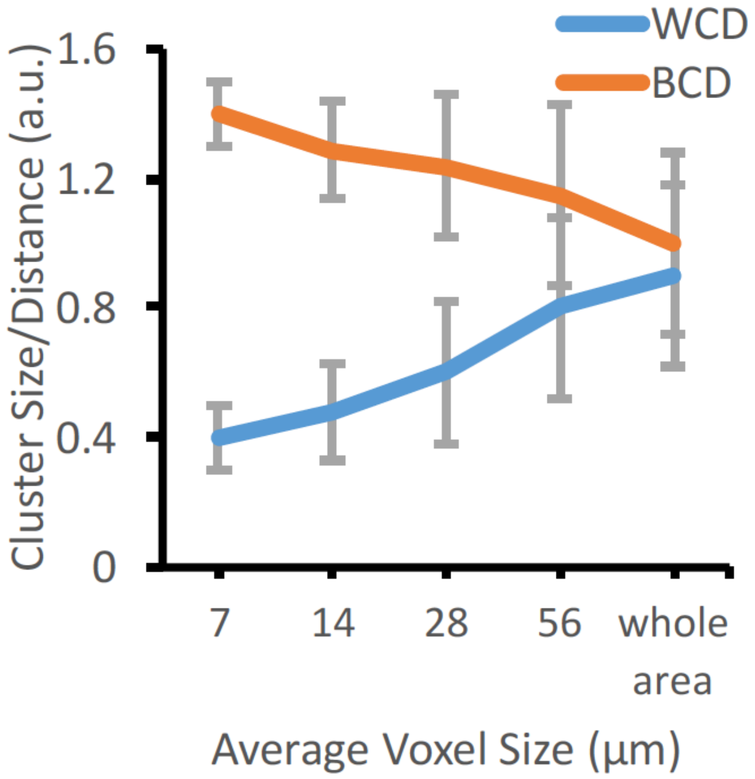

Supplementary Figure 13. Voxel size influences the resolution of drug clustering. To compare the quality of clusters, we measured two metrics: The distance between wild type cluster and mutant clusters (BCD, the larger the better), and the distance within wild type cluster (WCD, the smaller the better). We increased the size of our voxels (in average 14um, 28um, and 56um) to show the gradual degradation of clusters as the voxels get bigger and eventually each area is represented just by one voxel. Our results show both metrics degrade significantly when we use global pooling instead of voxel correlation: BCD decrease ~56% (from 1.62 to 0.91) and WCD increase ~110% (0.38 to 0.80). The graph represents mean ± SD (n= 20). Source data are provided as a Source Data file.

Supplementary Table 1: Compound library

Compound	CAS Number	Supplier (Catalog #)		Cluster	Primary Pathway(s)	Mechanism Details
Allopregnanolone	516-54-1	Sigma (P8887)	1 µM		GABA (+)	Positive allosteric modulator of the GABAA receptor; endogenous pregnane neurosteroid
Alprazolam	28981-97-7	Sigma (A8800)	100 µM		GABA (+)	Positive allosteric modulator of the GABAA receptor; benzodiazepine
Bromocriptine	22260-51-1	Sigma (B2134)	100 µM	C3	Dopamine (+)	Preferential agonist of the dopamine D2 receptor; additional agonistic activity on 5-HT and α-adrenergic receptors
Carbamazepine	298-46-4	Sigma (C4024)	100 µM	C1	Na+ channels (-)	Inhibits Na+ channels
Clobazam	22316-47-8	Sigma (C8414)	50 µM	C2	GABA (+)	Positive allosteric modulator of the GABAA receptor; benzodiazepine
Clonazepam	1622-61-3	Sigma (C1277)	50 µM	C1	GABA (+)	Positive allosteric modulator of the GABAA receptor; benzodiazepine
Diazepam	439-14-5	Sigma (D0899)	10 µM		GABA (+)	Positive allosteric modulator of the GABAA receptor; benzodiazepine
Duloxetine	136434-34-9	Sigma (SML0474)	1 µM	C3	Serotonin (+), NE/EPI (+)	Selective serotonin and norepinephrine reuptake inhibitor; weak inhibitor of dopamine reuptake
Fluoxetine	56296-78-7	Sigma (F132)	10 µM	C3	Serotonin (+)	Selective serotonin reuptake inhibitor
Fluvoxamine	61718-82-9	Sigma (F2802)	10 µM	C3	Serotonin (+)	Selective serotonin reuptake inhibitor
Haloperidol	52-86-8	Sigma (H1512)	10 µM		Dopamine (-)	Antagonist of the dopamine D2 receptor; additional targets may include 5-HT2, NMDA, and α-adrenergic receptors
L-701,324	142326-59-8	Sigma (L0258)	1 µM		Glutamate (-)	Antagonist of the NMDA glutamate receptor
Mepivacaine	1722-62-9	Sigma (M3189)	100 µM		Na+ channels (-)	Inhibits Na+ channels; local anesthetic
Methadone	1095-90-5	Sigma (M0267)	100 µM		Opiates (+)	Agonist of the µ-opioid receptor; may block the NMDA glutamate receptor; synthetic opioid
Metoprolol	56392-17-7	Sigma (M5391)	100 µM	C1	NE/EPI (-)	Antagonist of the β1-adrenergic receptor
Midazolam	59467-96-8	Tocris (2832)	10 µM		GABA (+)	Positive allosteric modulator of the GABAA receptor; benzodiazepine
Mifepristone	84371-65-3	Santa Cruz (203134)	25 µM	C1	Progesterone (-), Glucocorticoids (-)	Antagonist of the progesterone and glucocorticoid receptors
MPEP	219911-35-0	Sigma (M5435)	10 µM		Glutamate (-)	Antagonist of the metabotropic glutamate receptor 5
Pargyline	306-07-0	Sigma (P8013)	100 µM		Monoamines (+)	Inhibitor of monoamine oxidase B; may also inhibit monoamine oxidase A
Pergolide	66104-23-2	Sigma (P8828)	1 µM		Dopamine (+)	Agonist of the dopamine D2 receptor; additional agonistic activity at dopamine D3, α-adrenergic, and serotonin 5-HT receptors
Progesterone	57-83-0	Santa Cruz (296138)	0.4 µM	C1	Progestogen (+)	Agonist of the nuclear progesterone receptor; antagonist of the mineralocorticoid receptor; precursor of neurosteroids such as allopregnanolone
Promethazine	58-33-3	Sigma (P4651)	30 µM		Histamine (-)	Antagonist of the histamine H1 receptor; additional antagonistic activity at the muscarinic acetylcholine receptor
Pyrilamine	59-33-6	Sigma (P5514)	100 µM	C3	Histamine (-)	Antagonist of the histamine H1 receptor
Stiripentol	49763-96-4	Sigma (S6826)	20 µM		GABA (+)	Agonist of the GABAA receptor

Supplementary Table 2: Connectivity fingerprint ranking. Functional connectivity fingerprints from compound-treated scn1lab mutants have been ranked based on Euclidian distance from the wild-type sibling control cluster. Source data are provided as a Source Data file.

Compound	Rank	SD
Sibling (DMSO)	0.00	0.04
Mifepristone	0.22	0.09
Carbamazepine	0.26	0.06
Progesterone	0.32	0.37
Fluvoxamine	0.35	0.08
Clonazepam	0.41	0.92
Metoprolol	0.52	0.58
Bromocriptine	0.63	1.45
Clobazam	0.78	0.95
Fluoxetine	0.87	0.97
Pyrilamine	0.98	0.31
Allopregnanolone	1.30	1.31
Midazolam	1.52	1.19
Promethazine	1.61	0.82
Duloxetine	1.78	1.26
Alprazolam	1.82	0.83
Diazepam	1.86	0.91
Pargyline	1.92	0.76
Pergolide	2.01	0.91
Haloperidol	2.11	1.04
Mepivacaine	2.26	1.19
Pargyline	2.31	1.39
Pergolide	2.34	1.33
Methadone	2.37	1.52
L-701,324	2.38	1.90
MPEP	2.46	2.26
Stiripentol	3.92	1.50
Untreated (DMSO)	4.92	2.21

	Behavioral Metrics						Divergence from Baseline							
Condition	FV_{mean}	AV_{mean}	$HV_{\%}$	dTB_{mean}	TB_{mean}	AV_{STD}	FV_{mean}	AV_{mean}	$HV_{\%}$	dTB_{mean}	TB_{mean}	AV_{STD}	Efficacy	Side Effect
M (100) + F (100)	0.3196	0.2383	0.4344	0.4168	0.2372	0.3727	-0.6804	-0.7617	-0.5656	-0.5832	-0.7628	-0.6273	0.9599	0.6681
M (30) + F(30)	0.7534	0.6181	0.5806	0.6630	0.5206	0.6157	-0.2466	-0.3819	-0.4194	-0.3370	-0.4794	-0.3843	0.9107	0.3816
M (30) + F(100)	0.5609	0.4373	0.5562	0.4967	0.5104	0.5418	-0.4391	-0.5627	-0.4438	-0.5033	-0.4896	-0.4582	0.9100	0.4847
M (100) + F (30)	0.4314	0.4614	0.4531	0.5152	0.3969	0.5494	-0.5686	-0.5386	-0.5469	-0.4848	-0.6031	-0.4506	0.9091	0.5345
M (10) + F (100)	0.7011	0.5322	0.5810	0.4967	0.4807	0.5780	-0.2989	-0.4678	-0.4190	-0.5033	-0.5193	-0.4220	0.9086	0.4444
C (100) + F (100)	0.1076	0.1353	0.3396	0.3541	0.2673	0.4401	-0.8924	-0.8647	-0.6604	-0.6459	-0.7327	-0.5599	0.9002	0.7357
M (30) + C (100)	0.4303	0.3673	0.4097	0.3927	0.2904	0.5537	-0.5697	-0.6327	-0.5903	-0.6073	-0.7096	-0.4463	0.8795	0.5979
M (100) + C (100)	0.2509	0.1617	0.3758	0.4000	0.2655	0.4085	-0.7491	-0.8383	-0.6242	-0.6000	-0.7345	-0.5915	0.8604	0.6956
C (30) + F (100)	0.4810	0.4747	0.4125	0.4909	0.5252	0.5338	-0.5190	-0.5253	-0.5875	-0.5091	-0.4748	-0.4662	0.8506	0.5152
M (10) + C (100)	0.5600	0.4487	0.4498	0.4029	0.4792	0.5472	-0.4400	-0.5513	-0.5502	-0.5971	-0.5208	-0.4528	0.8303	0.5217
M (100) + C (30)	0.3389	0.2616	0.3763	0.4178	0.3588	0.4216	-0.6611	-0.7384	-0.6237	-0.5822	-0.6412	-0.5784	0.8199	0.6398
M (10) + F (30)	0.8305	0.6362	0.6649	0.6646	0.5918	0.6900	-0.1695	-0.3638	-0.3351	-0.3354	-0.4082	-0.3100	0.7912	0.3288
C (100) + F (30)	0.3597	0.2310	0.3651	0.3781	0.2804	0.4568	-0.6403	-0.7690	-0.6349	-0.6219	-0.7196	-0.5432	0.7712	0.6588
C (10) + F (100)	0.6103	0.4849	0.6937	0.5107	0.6230	0.6767	-0.3897	-0.5151	-0.3063	-0.4893	-0.3770	-0.3233	0.7508	0.4076
M (30) + C (30)	0.5485	0.4399	0.4477	0.4872	0.4031	0.6312	-0.4515	-0.5601	-0.5523	-0.5128	-0.5969	-0.3688	0.7101	0.5128
C (30) + F (30)	0.6106	0.4829	0.6288	0.5033	0.5698	0.6172	-0.3894	-0.5171	-0.3712	-0.4967	-0.4302	-0.3828	0.6792	0.4350
M (10) + C (30)	0.6604	0.5357	0.4863	0.4946	0.4730	0.6315	-0.3396	-0.4643	-0.5137	-0.5054	-0.5270	-0.3685	0.6402	0.4589
M (100) + C (10)	0.3613	0.3029	0.5054	0.4529	0.3504	0.5172	-0.6387	-0.6971	-0.4946	-0.5471	-0.6496	-0.4828	0.6291	0.5906
M (100) + F (10)	0.4707	0.5155	0.4839	0.6523	0.5314	0.5775	-0.5293	-0.4845	-0.5161	-0.3477	-0.4686	-0.4225	0.6119	0.4655
F (100)	0.7198	0.6341	0.6697	0.6475	0.7139	0.7090	-0.2802	-0.3659	-0.3303	-0.3525	-0.2861	-0.2910	0.6115	0.3194
C (100) + F (10)	0.3686	0.4978	0.4595	0.4496	0.3726	0.4979	-0.6314	-0.5022	-0.5405	-0.5504	-0.6274	-0.5021	0.6097	0.5615
C (10) + F (30)	0.7317	0.6218	0.7167	0.5570	0.7189	0.7228	-0.2683	-0.3782	-0.2833	-0.4430	-0.2811	-0.2772	0.6000	0.3285
M (30) + F(10)	0.7689	0.6219	0.6191	0.6687	0.5606	0.6971	-0.2311	-0.3781	-0.3809	-0.3313	-0.4394	-0.3029	0.5902	0.3502
F (30)	0.8487	0.7021	0.7344	0.7225	0.7173	0.7885	-0.1513	-0.2979	-0.2656	-0.2775	-0.2827	-0.2115	0.5200	0.2529
M (30) + C (10)	0.5609	0.4635	0.5159	0.5487	0.5467	0.6644	-0.4391	-0.5365	-0.4841	-0.4513	-0.4533	-0.3356	0.5193	0.4540
M (10) + C (10)	0.7005	0.6743	0.6478	0.6125	0.6375	0.6163	-0.2995	-0.3257	-0.3522	-0.3875	-0.3625	-0.3837	0.5085	0.3532
C (30) + F (10)	0.6405	0.5042	0.6470	0.5155	0.6036	0.6310	-0.3595	-0.4958	-0.3530	-0.4845	-0.3964	-0.3690	0.4891	0.4139
M (100)	0.6904	0.7632	0.5406	0.7908	0.5476	0.6110	-0.3096	-0.2368	-0.4594	-0.2092	-0.4524	-0.3890	0.4502	0.3565
C (100)	0.5709	0.5645	0.6778	0.4904	0.3273	0.5872	-0.4291	-0.4355	-0.3222	-0.5096	-0.6727	-0.4128	0.4410	0.4761
C (10) + F (10)	0.7599	0.7076	0.7365	0.6437	0.7218	0.7277	-0.2401	-0.2924	-0.2635	-0.3563	-0.2782	-0.2723	0.4186	0.2861
M (10) + F (10)	0.8839	0.6740	0.6702	0.7144	0.7206	0.8220	-0.1161	-0.3260	-0.3298	-0.2856	-0.2794	-0.1780	0.4105	0.2645
M (30)	0.8511	0.8251	0.6208	0.7443	0.6431	0.8535	-0.1489	-0.1749	-0.3792	-0.2557	-0.3569	-0.1465	0.4094	0.2617
F (10)	0.9907	0.8121	0.8419	0.7294	0.8862	0.8597	-0.0093	-0.1879	-0.1581	-0.2706	-0.1138	-0.1403	0.3710	0.1665
C (30)	0.7311	0.6180	0.7123	0.5371	0.7497	0.7501	-0.2689	-0.3820	-0.2877	-0.4629	-0.2503	-0.2499	0.3508	0.3267
M (10)	0.9599	0.8821	0.6874	0.7739	0.8898	0.8543	-0.0401	-0.1179	-0.3126	-0.2261	-0.1102	-0.1457	0.3305	0.1815
C (10)	0.8498	0.9116	0.7936	0.7211	0.7867	0.8214	-0.1502	-0.0884	-0.2064	-0.2789	-0.2133	-0.1786	0.3000	0.1950

Supplementary Table 3: Behavioral metrics, efficacy scores, and side effect scores from combinatorial screening. F, fluoxetine; M, Mifepristone; C, Clobazam. For each compound, the highest dose (100) corresponds to the concentration used in the initial light-sheet-based screen (see **Supplementary Table 1**). Compounds were also assessed at 30% and 10% of the highest concentration (30 and 10, respectively) individually and in all possible pairwise combinations (n=10 larvae per condition). Efficacy is calculated from local field potential recordings using an automated seizure detection algorithm. Behavioral metrics are detected under resting state conditions (i.e. in the absence of light stimuli) using automated locomotor tracking algorithms. Metrics are normalized to untreated (1% DMSO) age-matched mutant larvae and include the following parameters: mean forward swimming velocity (FV_{mean}; pixels s^{-1}), mean angular swimming velocity (AV_{mean}; pixels s^{-1}), percentage of time spent at high forward swimming velocity ($HV_{\%}$), mean tail bending angle (TB_{mean}; degrees), mean change in tail angle (dTB_m; degrees s^{-1}), and standard deviation of angular swimming velocity (AV_{STD}; pixels s^{-1}). Efficacy and Side Effect scores are calculated as described in **Methods**. Source data are provided as a Source Data file.

Sample	*p* -value (median)	Stationary Areas (%)
Wild-type 01	0.028	98.18
Wild-type 02	0.031	94.55
Wild-type 03	0.030	90.91
Wild-type 04	0.027	92.73
Wild-type 05	0.024	92.73
Wild-type 06	0.031	92.73
Wild-type 07	0.026	90.91
Wild-type 08	0.024	94.55
Wild-type 09	0.031	94.55
Wild-type 10	0.028	96.36
Mutant 01	0.031	94.55
Mutant 02	0.028	89.09
Mutant 03	0.027	92.73
Mutant 04	0.031	90.91
Mutant 05	0.032	94.55
Mutant 06	0.031	92.73
Mutant 07	0.025	96.36
Mutant 08	0.028	92.73
Mutant 09	0.027	85.45
Mutant 10	0.022	92.73

Supplementary Table 4: Functional connectivity between the majority of brain areas is stationary under resting-state conditions. GCaMP recordings from 10 scn1lab mutants and 10 wild-types were analyzed during the pre-stimulus resting state (i.e. the 10 minutes prior to the presentation of seizure-inducing light stimuli) using the Priestley-Subba Rao test of stationarity as described in **Methods**. The null hypothesis corresponds to dynamic (non-stationary) connectivity. Therefore, for $p<0.05$ the null hypothesis is rejected and the data are assumed to be stationary. For each larva, analysis is carried out on all 55 brain area pairs. For all 20 larvae analyzed, the median p-value of the second order stationarity test for the 55 area pairs was <0.05 [p-value (median) column]. On average, time series connectivity data from ~51 of 55 (93%) brain area pairs were found to be stationary ($p<0.05$; Stationary Areas column).

Area Name	Area Symbol	Average Segmentation Error (%)
**Cerebellum**	Ce	4.2
**Hindbrain left**	HBl	4.6
**Hindbrain right**	HBr	4.7
**Olfactory bulb left**	OBl	6.3
**Olfactory bulb right**	OBr	6.4
**Optical tectum, left**	OTl	5.4
**Optical tectum, right**	OTr	5.6
**Pallium**	Pa	16.2
**Sub-pallium**	Spa	17.2
**Habenula**	Ha	6.4
**Thalamus**	Th	8.5

Supplementary Table 5: Accuracy of brain segmentation. For each brain area in our analysis, we subtract the result of the segmentation algorithm from the manual segmentation and then normalize the difference to the size of the manual segmentation. Source data are provided as a Source Data file.
