# Supplementary material for: Engineering brain activity patterns by neuromodulator polytherapy for treatment of disorders
Source: Nat Commun. 2019 Jun 13;10:2620. doi: 10.1038/s41467-019-10541-1 (PMC6565674; doi:10.1038/s41467-019-10541-1)
Supplement: Supplementary file 3 — Reporting Summary [file 41467_2019_10541_MOESM3_ESM.pdf]

nature research

Corresponding author(s): DBPR, NCOMMS-18-28140B

Last updated by author(s): Apr 21, 2019

Reporting Summary

Nature Research wishes to improve the reproducibility of the work that we publish. This form provides structure for consistency and transparency in reporting. For further information on Nature Research policies, see Authors & Referees and the Editorial Policy Checklist .

Statistics

For all statistical analyses, confirm that the following items are present in the figure legend, table legend, main text, or Methods section.

n/a	Confirmed	
☐	☒	The exact sample size (n) for each experimental group/condition, given as a discrete number and unit of measurement
☐	☒	A statement on whether measurements were taken from distinct samples or whether the same sample was measured repeatedly
☐	☒	The statistical test(s) used AND whether they are one- or two-sided<br>*Only common tests should be described solely by name; describe more complex techniques in the Methods section.*
☒	☐	A description of all covariates tested
☐	☒	A description of any assumptions or corrections, such as tests of normality and adjustment for multiple comparisons
☐	☒	A full description of the statistical parameters including central tendency (e.g. means) or other basic estimates (e.g. regression coefficient) AND variation (e.g. standard deviation) or associated estimates of uncertainty (e.g. confidence intervals)
☒	☐	For null hypothesis testing, the test statistic (e.g. F, t, r) with confidence intervals, effect sizes, degrees of freedom and P value noted<br>*Give P values as exact values whenever suitable.*
☒	☐	For Bayesian analysis, information on the choice of priors and Markov chain Monte Carlo settings
☒	☐	For hierarchical and complex designs, identification of the appropriate level for tests and full reporting of outcomes
☐	☒	Estimates of effect sizes (e.g. Cohen's d, Pearson's r), indicating how they were calculated

Our web collection on statistics for biologists contains articles on many of the points above.

Software and code

Policy information about availability of computer code

Data collection	1) Labview source code used for calcium imaging data is available in https://github.com/rezaie99/NN-A59900-2
Data analysis	1) Python source code for motion correction and timeline extraction of images, 2) Bash files to run external tools (ANT) for registration, 3) Python code for frequency and synchronization analysis, 4) Python code for LFP analysis and correlation, and 5) MATLAB code for cluster analysis and statistical calculation. The source code are available in https://github.com/rezaie99/NN-A59900-2

For manuscripts utilizing custom algorithms or software that are central to the research but not yet described in published literature, software must be made available to editors/reviewers. We strongly encourage code deposition in a community repository (e.g. GitHub). See the Nature Research guidelines for submitting code & software for further information.

Data

Policy information about availability of data

All manuscripts must include a data availability statement. This statement should provide the following information, where applicable:

- Accession codes, unique identifiers, or web links for publicly available datasets
- A list of figures that have associated raw data
- A description of any restrictions on data availability

Data availability.
The data that support the findings of this study are available from the corresponding author upon request.

Field-specific reporting

Please select the one below that is the best fit for your research. If you are not sure, read the appropriate sections before making your selection.

☒ Life sciences ☐ Behavioural & social sciences ☐ Ecological, evolutionary & environmental sciences

For a reference copy of the document with all sections, see nature.com/documents/nr-reporting-summary-flat.pdf

Life sciences study design

All studies must disclose on these points even when the disclosure is negative.

Sample size

For screening using functional connectivity fingerprints (Fig. 2c) we report "A minimum of 5 larvae are analyzed per compound. This sample size is chosen to ensure sufficient statistical power to separate mutant larvae (magenta text) from sibling controls (cyan text; unpaired Student's t-test, $p < 0.01$; normality of datasets was tested using the Jarque–Bera normality test)" This is found in the LEGEND for Fig. 2. For testing the efficacy and side effects of polytherapy drug candidates (Fig. 3) we report "Scores have been normalized to untreated age-matched larvae (n=5+ larvae per condition for efficacy; n=10 larvae per condition for side effect)." This is found in the LEGEND for Fig. 3. For analyzing the effect of polytherapy drug candidates on functional connectivity (Fig. 4), we use report "10 larvae were analyzed per condition." This is found in the LEGEND for Fig. 4.

Data exclusions

NA

Replication

As noted above, for screening using functional connectivity fingerprints (Fig. 2c) we use a minimum of 5 larvae are analyzed per compound, each of which functions as a biological replicate. As is typical of most screens, initial hits were then retested to confirm activity using follow-up assays as detailed in Fig. 3 and Fig. 4.

Randomization

Zebrafish larvae are assigned to either a "mutant" or "wild-type" group based on the status of the scn1lab gene. As indicated in METHODS this is determined either by PCR genotyping or visual inspection to detect an associated pigmentation phenotype arising from loss of scn1lab. Larvae with a given genotype were randomly assigned to test compounds.

Blinding

As indicated in RESULTS and METHODS, functional connectivity analysis, seizure scores, and behavioral analysis are all done using automated algorithms that process recordings identically regardless of group/experimental condition. See the final paragraph of the "Brain-wide imaging reveals abnormal functional connectivity in epileptic mutants" section in RESULTS and the "Image processing and cluster analysis", "Behavioral analysis", and "LFP recording and analysis" sections in METHODS.

Reporting for specific materials, systems and methods

We require information from authors about some types of materials, experimental systems and methods used in many studies. Here, indicate whether each material, system or method listed is relevant to your study. If you are not sure if a list item applies to your research, read the appropriate section before selecting a response.

Materials & experimental systems

n/a	Involved in the study
☒	☐ Antibodies
☒	☐ Eukaryotic cell lines
☒	☐ Palaeontology
☐	☒ Animals and other organisms
☒	☐ Human research participants
☒	☐ Clinical data

Methods

n/a	Involved in the study
☒	☐ ChIP-seq
☒	☐ Flow cytometry
☒	☐ MRI-based neuroimaging

Animals and other organisms

Policy information about studies involving animals; ARRIVE guidelines recommended for reporting animal research

Laboratory animals

5dpf Zebrafish larva is used in all the experiments. The scn1labs552 line (also known as double indemnity or didy) has been described previously13 and was crossed onto a line expressing the genetically encoded calcium indicator GCaMP5G35 under the control of the pan-neuronal elavl3/HuC promoter [Tg(HuC:GCaMP5G); a generous gift of A Schier, Harvard, Cambridge, MA]. Homozygous mutant scn1labs552 larvae and age-matched siblings controls expressing the GCaMP5G reporter were obtained by crossing Tg(HuC:GCaMP5+/+; scn1lab+/-) adults.

Wild animals

n/a

Field-collected samples

n/a

Ethics oversight

n/a

Note that full information on the approval of the study protocol must also be provided in the manuscript.
