# Supplementary material for: Engineering brain activity patterns by neuromodulator polytherapy for treatment of disorders
Source: Nat Commun. 2019 Jun 13;10:2620. doi: 10.1038/s41467-019-10541-1 (PMC6565674; doi:10.1038/s41467-019-10541-1)
Supplement: Supplementary file 4 — Description of Additional Supplementary Files [file 41467_2019_10541_MOESM4_ESM.pdf]

Description of Additional Supplementary Files

File Name: Supplementary Movie 1
Description: **Light-triggered seizure-like locomotor activity in scn1lab mutant larvae**. Videos show light-triggered locomotor activity in scn1labs552 homozygous mutants and in 96-well plates. Locomotor activity was recorded in darkness using infrared illumination and an automated tracking platform. Light stimuli consist of two brief (500 ms) light pulses separated by a 1 second interval. In each video, eleven wells contain individual zebrafish larvae while the dark well located in the bottom right position contains a fluorescent dye that is sensitive to illumination in the visible spectrum and serves to indicate the timing of the light stimuli.

File Name: Supplementary Movie 2
Description: **Light-triggered seizure-like locomotor activity in sibling control larvae.** Videos show light-triggered locomotor activity in age-matched sibling controls in 96-well plates. Locomotor activity was recorded in darkness using infrared illumination and an automated tracking platform. Light stimuli consist of two brief (500 ms) light pulses separated by a 1 second Page 2/3 interval. In each video, eleven wells contain individual zebrafish larvae while the dark well located in the bottom right position contains a fluorescent dye that is sensitive to illumination in the visible spectrum and serves to indicate the timing of the light stimuli.

File Name: Supplementary Movie 3
Description: **Light-triggered seizure-like GCaMP activity in scn1lab mutant larvae, example 1.** Videos show light-triggered GCaMP activity from a single z-plane in scn1labs552 homozygous mutants. GCaMP activity was imaged as described using our high-throughput light sheet platform. Light stimuli consist of two brief (500 ms) light pulses separated by a 1 second interval. The beginning of each video corresponds to the presentation of the light stimulus.

File Name: Supplementary Movie 4
Description: **Light-triggered seizure-like GCaMP activity in scn1lab mutant larvae, example 2.** Videos show light-triggered GCaMP activity from a single z-plane in scn1labs552 homozygous mutants. GCaMP activity was imaged as described using our high-throughput light sheet platform. Light stimuli consist of two brief (500 ms) light pulses separated by a 1 second interval. The beginning of each video corresponds to the presentation of the light stimulus.

File Name: Supplementary Movie 5
Description: **Light-triggered seizure-like GCaMP activity in sibling control larvae, example 1.** Videos show light-triggered GCaMP activity from a single z-plane in age-matched sibling controls. GCaMP activity was imaged as described using our high-throughput light sheet platform. Light stimuli consist of two brief (500 ms) light pulses separated by a 1 second interval. The beginning of each video corresponds to the presentation of the light stimulus.

File Name: Supplementary Movie 6
Description: **Light-triggered seizure-like GCaMP activity in sibling control larvae, example 2.** Videos show light-triggered GCaMP activity from a single z-plane in age-matched sibling controls. GCaMP activity was imaged as described using our high-throughput light sheet platform. Light stimuli consist of two brief (500 ms) light pulses separated by a 1 second interval. The beginning of each video corresponds to the presentation of the light stimulus.

File Name: Supplementary Movie 7

Description: **Spontaneous locomotor activity in scn1lab mutant larvae after optimal polytherapy.** The mutants are treated simultaneously with fluoxetine and mifepristone using one of the optimal polytherapy dose regimens (both drugs at 30% of dosage).
